# Supplementary figures and images for: Expression of the ACE2 Virus Entry Protein in the Nervus Terminalis Reveals the Potential for an Alternative Route to Brain Infection in COVID-19
Source: Front Cell Neurosci. 2021 Jul 5;15:674123. doi: 10.3389/fncel.2021.674123 (PMC8287262; doi:10.3389/fncel.2021.674123)

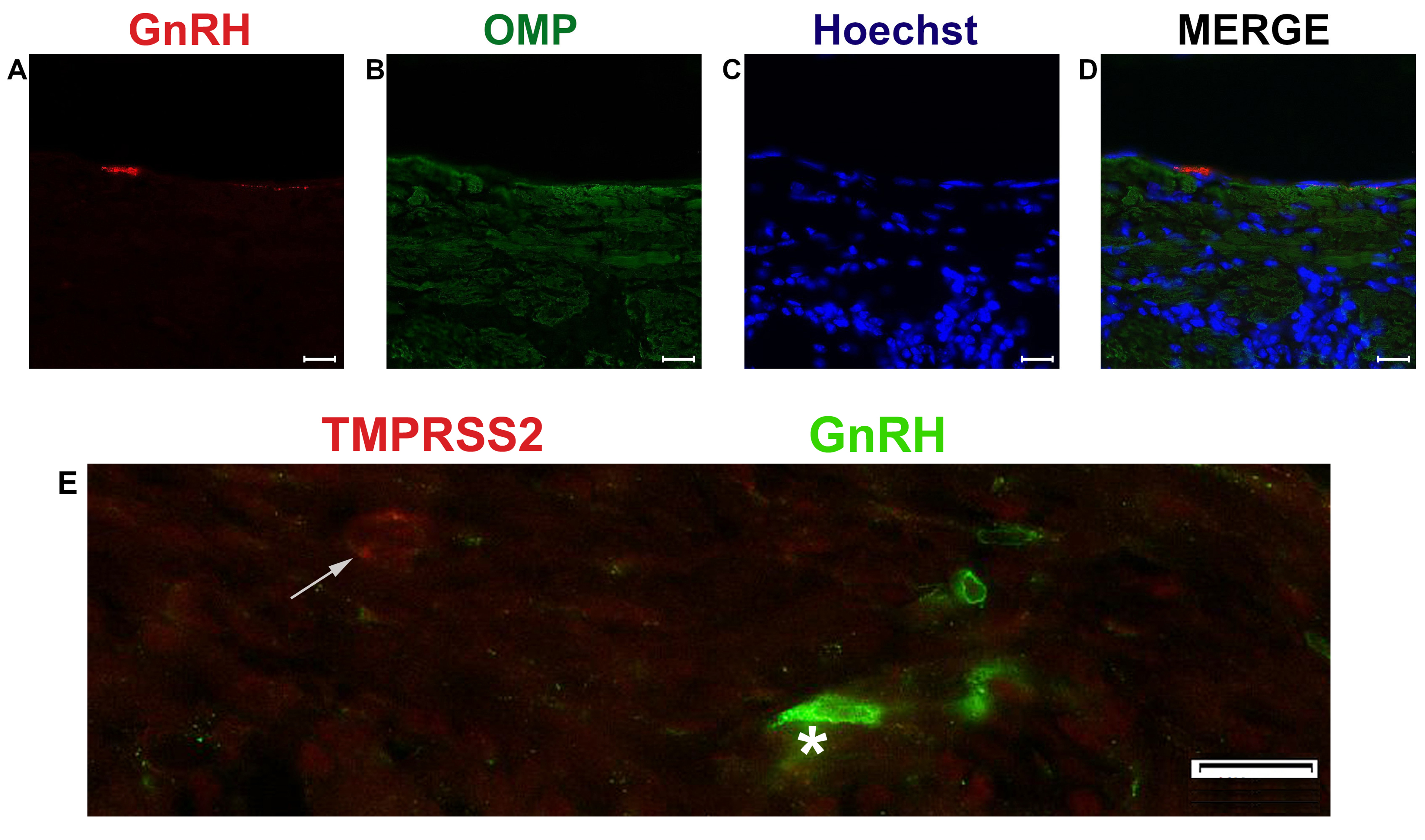

Supplement: Supplementary Figure 1 — Examples of double immunofluorescent labeling of nervus terminalis neurons with the markers GnRH and olfactory marker protein (OMP) (A–D), and GnRH and TMPRSS2 (E). Label for GnRH (A) and OMP (B) in the medial region adjacent to the olfactory bulbs as indicated in Figure 1. Nuclei are stained with Hoechst 33258 (C) and the merged image is shown in (D). GnRH-labeled cells were never labeled for OMP in this region. (E) GnRH-labeled nervus terminalis neurons (one neuron indicated with the white asterisk) did not co-localize with cells positive for TMPRSS2 (white arrow). Scale bars: 20 μm. [file Image_1.JPEG]
