# Supplementary material for: Expression of the ACE2 Virus Entry Protein in the Nervus Terminalis Reveals the Potential for an Alternative Route to Brain Infection in COVID-19
Source: Front Cell Neurosci. 2021 Jul 5;15:674123. doi: 10.3389/fncel.2021.674123 (PMC8287262; doi:10.3389/fncel.2021.674123)
Supplement: Supplementary Table 1 — Primary and secondary antibodies used in this study. ACE2, angiotensin converting enzyme 2; CHAT, choline acetyltransferase; GnRH1, gonadotropin releasing hormone 1; OMP, olfactory marker protein; TMPRSS2, transmembrane protease serine 2. [file Table_1.DOCX]

**SUPPLEMENTAL TABLES**

**Table S1**. Primary and secondary antibodies used in this study.

| **Primary antibodies** | **Company** | **Catalog #** | **Type** |
| --- | --- | --- | --- |
| ACE2 | R&D Systems | AF3437 | goat polyclonal |
| ACE2 | ABclonal | A4612 | rabbit monoclonal |
| Cathepsin B | R&D Systems | AF965 | goat polyclonal |
| Cathepsin L | R&D Systems | AF1515 | goat polyclonal |
| CHAT | Proteintech | 24418-1-AP | rabbit polyclonal |
| GnRH1 | Proteintech | 26950-1-AP | rabbit polyclonal |
| OMP | WAKO | 544-10001 | goat polyclonal |
| TMPRSS2 | Novus Biologicals | NBP3-00492 | rabbit monoclonal |
| TMPRSS2 | Abcam | ab242384 | rabbit monoclonal |
| TMPRSS2 | St John’s | STJ11102428 | rabbit monoclonal |
| **Secondary antibodies** | | | **Fluorescent conjugate** |
| donkey anti-rabbit | Abcam | ab15006 | Alexa Fluor 488 |
| donkey anti-goat | Abcam | ab150136 | Alexa Fluor 594 |
| horse anti-rabbit | Vector | DI-1094 | Alexa Fluor 594 |
| horse anti-goat | Vector | DI-3088 | Alexa Fluor 488 |

Abbreviations: ACE2, angiotensin converting enzyme 2; CHAT, choline acetyltransferase; GnRH1, gonadotropin releasing hormone 1; OMP, olfactory marker protein; TMPRSS2, transmembrane protease serine 2.
