# Supplementary material for: Expression of the ACE2 Virus Entry Protein in the Nervus Terminalis Reveals the Potential for an Alternative Route to Brain Infection in COVID-19
Source: Front Cell Neurosci. 2021 Jul 5;15:674123. doi: 10.3389/fncel.2021.674123 (PMC8287262; doi:10.3389/fncel.2021.674123)
Supplement: Supplementary Table 2 — Numbers of animals, sections, and numbers of neurons double-labeled for nervus terminalis markers (GnRH, CHAT) and ACE2. [file Table_2.DOCX]

**TABLE S2.** Numbers of animals, sections, and numbers of neurons double-labeled for nervus terminalis markers (GnRH, CHAT) and ACE2.

|  | **# of sections** | **# of GnRH neurons** | **# of GnRH and ACE2** | **%** |
| --- | --- | --- | --- | --- |
| **GnRH and ACE2** | |  |  |  |
| Mouse 1 | 16 | 25 | 23 | 92.0 |
| Mouse 2 | 21 | 30 | 27 | 90.0 |
| Mouse 3 | 15 | 21 | 19 | 90.5 |
| Mouse 4 | 11 | 10 | 8 | 80.0 |
| Mouse 5 | 22 | 33 | 30 | 90.9 |
| **Sum/mean** | **85** | **119** | **107** | **89.9** |
|  |  |  |  |  |
| **CHAT and ACE2** | | |  |  |
| Mouse 1 | 21 | 20 | 2 | 10.0 |
| Mouse 2 | 15 | 18 | 2 | 11.1 |
| Mouse 3 | 18 | 14 | 1 | 7.1 |
| **Sum/mean** | **54** | **52** | **5** | **9.4** |
|  |  |  |  |  |
| **t-test** |  |  |  | **p<0.0001** |
